# Pasteurella Empyema as a Complication of Postoperative Pleural Effusion Following Coronary Artery Bypass Grafting (CABG)

**DOI:** 10.7759/cureus.115074

**Published:** 2026-08-24

**Authors:** Zaineb Ahmad, Manveer Sandhu, Milan Patel, Manni Golbarg, Kyle Sharron, Gabrielle Brini, John Ferguson

**Affiliations:** 1 Internal Medicine, Touro College of Osteopathic Medicine, Middletown, USA; 2 Neurology, Touro College of Osteopathic Medicine, Middletown, USA; 3 Biomedical Sciences, Touro College of Osteopathic Medicine, Middletown, USA; 4 Emergency Medicine, Touro College of Osteopathic Medicine, Middletown, USA; 5 Pulmonology, Garnet Health Medical Center, Middletown, USA

**Keywords:** bacterial empyema, empyema, infectious disease, pasteurella multocida, pulmonology

## Abstract

A 60-year-old male with a history of chronic obstructive pulmonary disease (COPD) and prior lung malignancy with radiation therapy presented with progressive dyspnea status post coronary artery bypass grafting (CABG) five weeks postoperative. Initial workup demonstrated bilateral pleural effusions that were originally attributed to postoperative complications, though thoracocentesis demonstrated hemorrhagic fluid. Pleural cultures were positive for *Pasteurella *species, confirming empyema consistent with a history of household exposure to domesticated cats, dogs, and turtles. The inpatient course was characterized by rapid deterioration with bacteremia and acute hypoxic respiratory failure, requiring mechanical ventilation. This case demonstrates that respiratory transmission of *Pasteurella multocida* in immunocompromised individuals can cause life-threatening, invasive disease without direct inoculation, emphasizing the importance of zoonotic pathogens and obtaining relevant exposure histories when evaluating pleural infections in immunocompromised populations.

## Introduction

Empyema is the presence of purulent, bacteria-laden exudate in the pleural space, most often related to pneumonia, chest trauma, or thoracic surgery, and carries substantial morbidity and mortality if not recognized and treated promptly [1,2]. Recognized risk factors include chronic lung disease, cardiovascular disease, diabetes mellitus, gastroesophageal reflux disease, conditions predisposing to aspiration, and immunocompromised states [1,3]. Diagnosis relies on contrast-enhanced computed tomography and ultrasound, which are more sensitive than plain radiography, together with diagnostic thoracentesis; pleural fluid typically demonstrates a low pH (<7.2), low glucose (<60 mg/dL), and elevated lactate dehydrogenase [1]. *Staphylococcus aureus*,* *including methicillin-resistant strains, is the most commonly identified organism, followed by streptococcal species and a range of Gram-negative bacteria, though cultures remain negative in a substantial proportion of cases [1,4].

Although *Pasteurella multocida*, a facultatively anaerobic, Gram-negative coccobacillus found in the oral flora of cats and dogs, is most commonly associated with animal bites and scratches, invasive pulmonary and pleural infections can occur without any reported traumatic inoculation, presumably via inhalation of contaminated aerosols or contact with the secretions of colonized animals [5,6]. Patients with comorbid cardiopulmonary disease such as chronic obstructive pulmonary disease (COPD) are at increased risk, and *P. multocida *empyema can be fatal, underscoring the need for prompt recognition [6,7]. Such infections may be especially difficult to identify when pleural effusions are initially attributed to recent cardiothoracic surgery. This report describes *P. multocida *empyema with bacteremia in a patient with COPD and recent coronary artery bypass grafting (CABG), suggesting that *P. multocida *infection may occur in the absence of a bite or scratch and should be considered in vulnerable patients with relevant animal exposure, and highlights the importance of obtaining a detailed animal exposure history.

## Case presentation

A 60-year-old male with a history of coronary artery disease status post CABG five weeks earlier, COPD, recurrent pleural effusions, and previously treated stage III squamous cell carcinoma of the left lung presented with progressively worsening dyspnea. His oncologic history was notable for a 7.8 × 6.9 cm aortopulmonary-window mass diagnosed by endobronchial ultrasound-guided biopsy. The tumor had a PET standardized uptake value of 18.5. He subsequently underwent concurrent chemoradiation with carboplatin and etoposide, completing 4,400 cGy, followed by durvalumab immunotherapy.

The patient's social history was notable for substantial household animal exposure. He lived with two dogs, two cats, and two turtles. He denied any recent animal bites, scratches, or other traumatic inoculation. During the month preceding admission, he developed progressively worsening dyspnea. He denied chills, nausea, vomiting, chest pain, peripheral edema, or weight loss. His respiratory symptoms eventually became severe enough to require helicopter transfer to a higher level of care.

On arrival, the patient was febrile and in acute respiratory distress. Temperature was 38.4°C (101.1°F), with a heart rate of 118 beats/min, respiratory rate of 32 breaths/min, and blood pressure of 110/75 mmHg. Oxygen saturation was 88% on room air and improved only to 91% with 4 L/min oxygen via nasal cannula. He reported a congested cough with intermittent hemoptysis. Initial laboratory studies demonstrated a white blood cell count of 26.3 × 10⁹/L, lactate of 4.7 mmol/L, creatinine of 1.3 mg/dL, blood urea nitrogen (BUN) of 19 mg/dL, platelet count of 162 × 10⁹/L, and D-dimer of 2,749 ng/mL (Table 1).

**Table 1:** Clinical course, laboratory findings, diagnostic evaluation, and treatment BUN: blood urea nitrogen; CTA: computed tomography angiography; LDH: lactate dehydrogenase; PE: pulmonary embolism

Hospital Day	Clinical status	Vital signs/laboratory findings	Microbiology/pleural findings	Intervention/outcome
Day 1	Progressive dyspnea, cough, hemoptysis, acute respiratory distress	T 38.4°C; HR 118; RR 32; BP 110/75; SpO₂ 88% RA, 91% on 4 L NC; WBC 26.3 × 10⁹/L; lactate 4.7; Cr 1.3; BUN 19; platelets 162 × 10⁹/L; D-dimer 2,749	—	CTA negative for PE; bilateral effusions R>L, atelectasis and pulmonary edema; BiPAP; ceftriaxone + metronidazole
Day 2	Persistent respiratory distress	Pleural pH 7.0; glucose 29; protein 3.6 g/dL; LDH 847; nucleated cells 165,000; neutrophils 92%	Gram-negative coccobacilli; 2 pleural specimens positive for P. multocida; 2 blood-culture sets positive for P. multocida	875 mL hemorrhagic fluid removed; right chest tube placed; antibiotics changed to Unasyn 3 g IV q6h
Days 3-4	Persistent infection followed by acute deterioration	—	—	Continued Unasyn and pleural drainage
Day 4	Worsening hypoxia, tachycardia, diaphoresis, obtundation	—	—	ICU transfer and intubation for acute hypoxic respiratory failure
Within 72 h of Unasyn	Microbiologic improvement	—	Blood cultures cleared	Continued targeted therapy
Day 7	Clinical improvement	—	—	Chest tube removed; no additional drainage required
Day 12	Respiratory recovery	—	—	Extubated; no vasopressors required
Day 14	Completion of antimicrobial therapy	—	—	Completed 14-day antimicrobial course
Day 16	Continued clinical recovery	—	—	Discharged after 16-day hospitalization
Final Outcome	Recovered	—	Follow-up imaging unavailable	Recovery following ICU care, antimicrobial therapy, and pleural drainage

**Figure 1:**
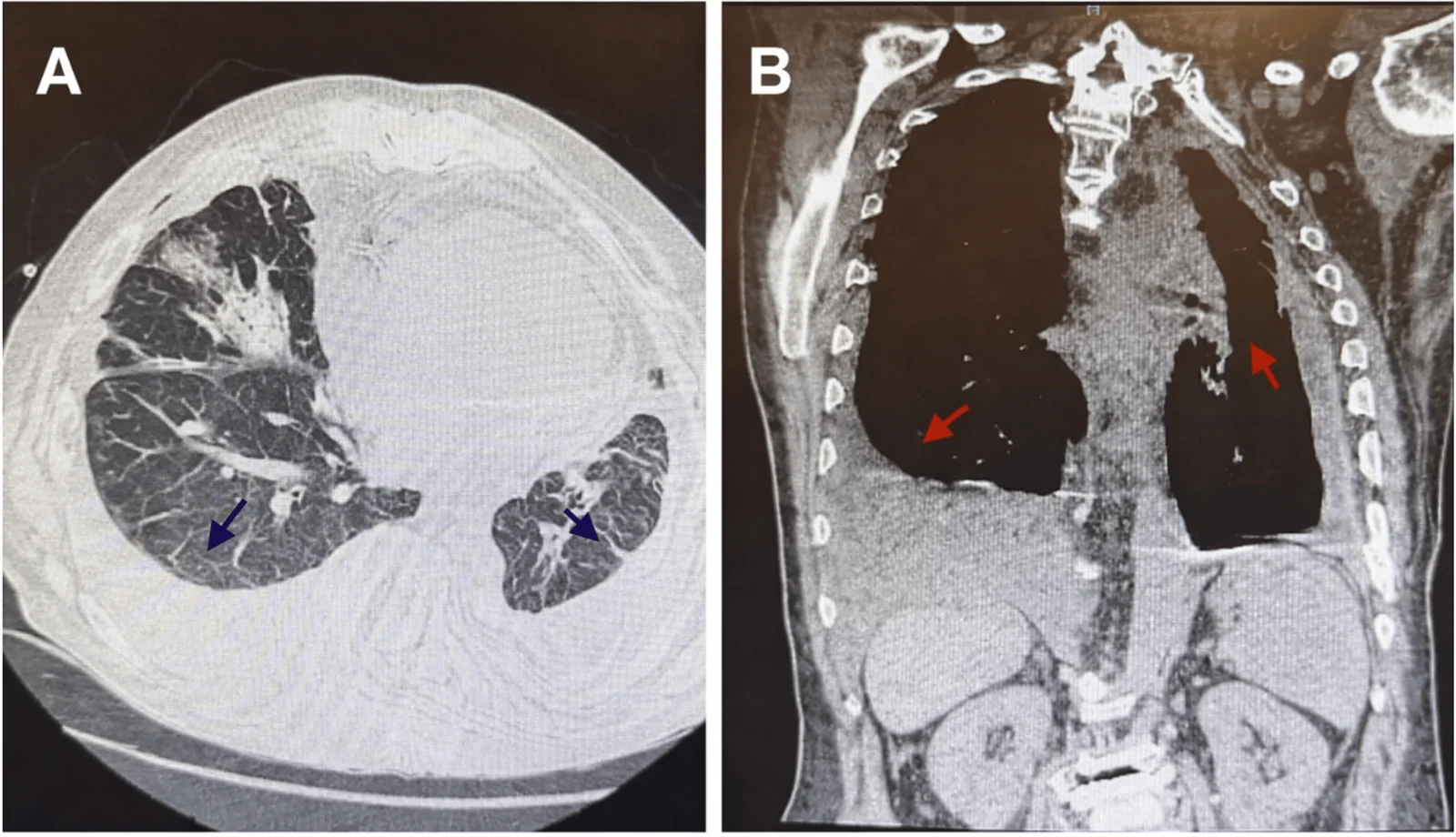
CT angiography of the chest obtained on hospital Day 1 demonstrating no acute pulmonary embolism (A) Axial image showing moderate bilateral pleural effusions, greater on the right, with adjacent compressive atelectatic change and pulmonary edema. (B) Coronal image demonstrating similar findings. Exclusion of pulmonary embolism redirected evaluation toward the pleural process, which was subsequently confirmed to represent Pasteurella multocida empyema.

Given his acute hypoxemia, tachycardia, recent major surgery, and markedly elevated D-dimer, pulmonary embolism was considered in the initial differential diagnosis. CT angiography of the chest was therefore obtained and demonstrated no acute pulmonary embolism. Instead, imaging revealed moderate bilateral pleural effusions, greater on the right, with adjacent compressive atelectatic changes and pulmonary edema (Figure 1).

Given the patient's recent CABG and history of recurrent pleural effusions, the initial working diagnosis favored a postoperative pleural effusion, including a possible post-pericardiotomy inflammatory process. However, his fever, marked leukocytosis, elevated lactate, progressive respiratory symptoms, and persistent pleural abnormalities raised concern for a superimposed infectious process. He subsequently required bilevel positive airway pressure (BiPAP) support because of worsening respiratory distress.

**Figure 2:**
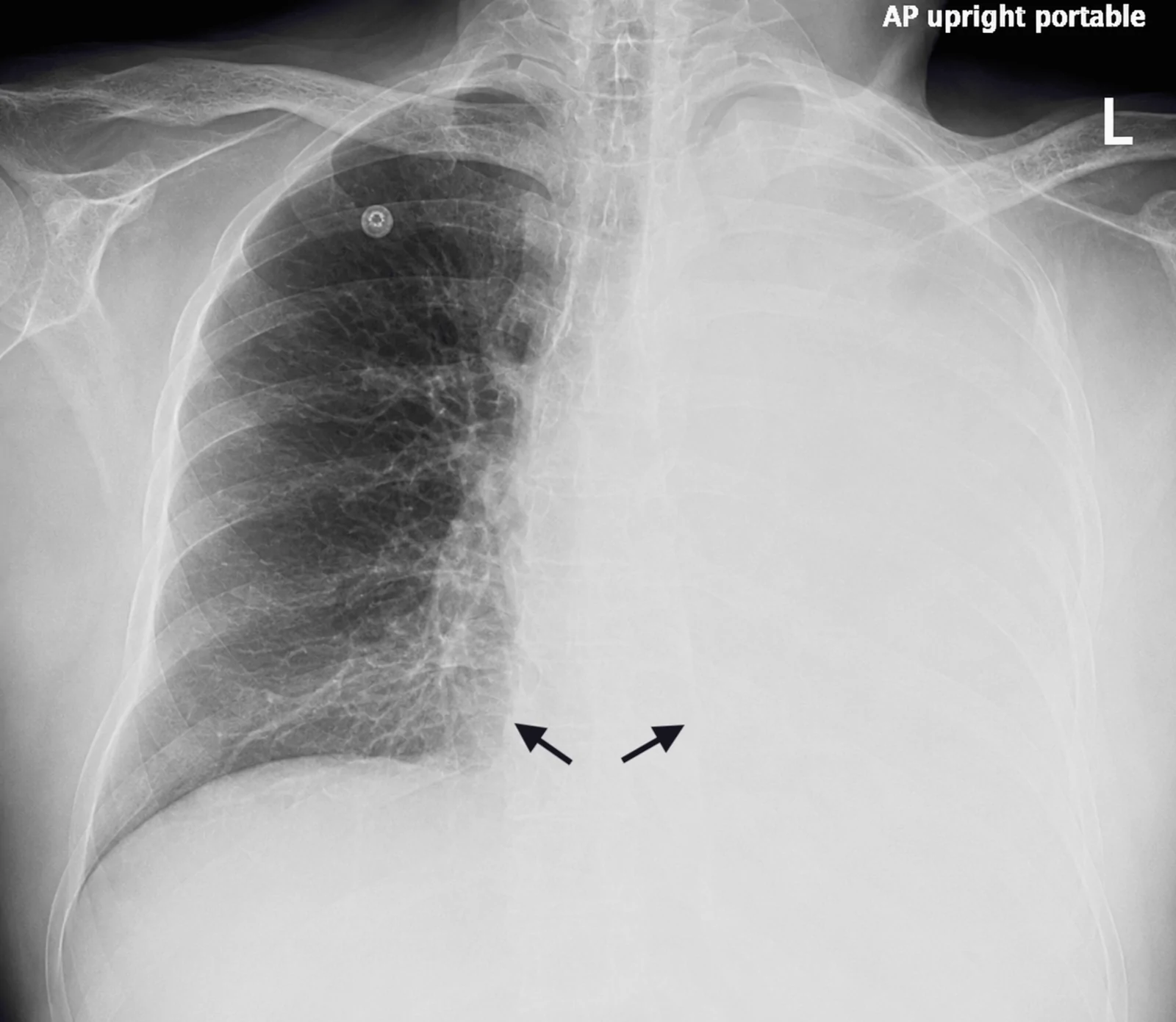
Chest radiograph obtained on hospital Day 2 Demonstrating persistent large left and moderate right pleural effusions with near-complete collapse/consolidation of the left lung. Persistent pleural abnormalities in the setting of fever, leukocytosis, and progressive respiratory distress prompted diagnostic thoracentesis.

Chest radiography demonstrated persistent large left and moderate right pleural effusions with near-complete collapse/consolidation of the left lung (Figure 2). Diagnostic thoracentesis yielded 875 mL of hemorrhagic pleural fluid with partial symptomatic improvement. Pleural-fluid analysis demonstrated a pH of 7.0, glucose of 29 mg/dL, protein of 3.6 g/dL, lactate dehydrogenase (LDH) of 847 U/L, total nucleated cell count of 165,000 cells, and 92% neutrophils (Table 1). Concurrent serum protein was 6.7 g/dL, and serum LDH was 195 U/L. The pleural-fluid-to-serum protein ratio was 0.54, while the pleural-fluid-to-serum LDH ratio was 4.34, satisfying two of Light's criteria for an exudative effusion. The markedly low pleural-fluid pH and glucose, substantial neutrophilic predominance, and high nucleated-cell count were strongly suggestive of complicated bacterial pleural infection. Cytology was negative for malignancy.

Gram stain of the pleural fluid demonstrated Gram-negative coccobacilli. Two separate pleural-fluid specimens subsequently grew *P. multocida*. Two separate blood-culture sets also grew *P. multocida*, establishing concurrent empyema and bacteremia. Antimicrobial susceptibility testing demonstrated susceptibility to penicillin G, ampicillin, and ceftriaxone; intermediate susceptibility to doxycycline and ciprofloxacin; and resistance to gentamicin. Methicillin-resistant *Staphylococcus aureus *(MRSA) nasal polymerase chain reaction (PCR) was positive, consistent with colonization, but there was no additional clinical or microbiologic evidence establishing MRSA as the causative pathogen.

Empiric antimicrobial therapy with intravenous ceftriaxone and metronidazole was initiated on admission. Following identification of *P. multocida *in the pleural fluid and blood, Infectious Disease was consulted, and therapy was transitioned to intravenous ampicillin-sulbactam (Unasyn) 3 g every six hours. The patient received a total of 14 days of antimicrobial therapy. Repeat blood cultures cleared within 72 hours of initiation of Unasyn, demonstrating microbiologic resolution of the bacteremia.

For pleural source control, a right-sided chest tube was placed on hospital Day 2. Daily chest-tube output measurements were not available in the records reviewed. The chest tube remained in place until hospital Day 7 and was subsequently removed. No additional pleural drainage procedures or surgical interventions were required (Table 1).

Despite antimicrobial therapy and pleural drainage, the patient experienced acute clinical deterioration on hospital Day 4, with worsening hypoxia, tachycardia, diaphoresis, and obtundation. He was transferred to the intensive care unit and underwent emergent endotracheal intubation for acute hypoxic respiratory failure. He remained in the ICU for 12 days and required mechanical ventilation during this period. No vasopressor support was required (Table 1).

The patient was successfully extubated on hospital Day 12. His respiratory status and overall clinical condition subsequently improved. Follow-up chest imaging was not available for review. The patient ultimately recovered from the acute infection and respiratory failure and was discharged after a total hospital stay of 16 days (Table 1).

## Discussion

This case brings together several instructive features of invasive *P. multocida *infection acquisition without a reported bite or scratch, presentation as empyema with concurrent bacteremia, an initial picture that closely mimicked a postoperative effusion, and rapid progression to respiratory failure in a host whose pulmonary defenses were compromised by chronic disease and prior therapy.

Although most human pasteurellosis follows direct inoculation, a substantial proportion of invasive infections arise without traumatic injury, presumably through inhalation of aerosolized secretions or mucosal contact with colonized animals, whose oral and nasal carriage rates reach 60-90% in cats and roughly 50-55% in dogs [6]. In a comparative series, non-bite/scratch pasteurellosis presented predominantly as pneumonia and bacteremia, occurred almost exclusively in patients with moderate-to-severe comorbidities (100% vs 14% in bite/scratch cases, p < 0.001), and behaved as an opportunistic infection that sometimes occurred even without identifiable recent animal contact [4]. Consistent with this, the respiratory tract is the second most common site of human *P. multocida *infection, and pulmonary isolates disproportionately represent the *P. multocida *subsp. multocida subgroup [8]. Airway recovery of the organism may at times reflect commensal colonization of an already abnormal respiratory tract, but serious infection pneumonia, lung abscess, and empyema is well described in patients with underlying pulmonary disease [5,9]. Against this background, the patient's substantial household exposure to dogs and cats is the most plausible source, although the exact route of acquisition cannot be established from a single case.

Why this ordinarily indolent commensal produced invasive disease is best explained by the patient's impaired pulmonary defenses. His COPD and history of thoracic irradiation likely diminished local host defense and may have contributed to susceptibility to invasive lower respiratory tract infection. COPD entails broad, smoking-dependent suppression of innate lung defense, impaired mucociliary clearance, reduced surfactant proteins A and D, defective alveolar macrophage phagocytosis, and diminished Toll-like receptor expression that favors pathogen colonization and progression to infection, compounded by adaptive immune dysfunction including regulatory and exhausted T-cell populations [10-12]. Prior chemoradiation and immunotherapy for NSCLC added local structural injury and altered clearance, plausibly lowering the threshold for an animal oral commensal to establish invasive pleuropulmonary disease. This is consistent with the wider literature identifying malignancy, chronic respiratory disease, and related comorbid states as recurrent predisposing conditions for systemic pasteurellosis [6,7].

The early course also illustrates a common diagnostic pitfall. Pleural effusions are frequent after cardiac surgery, and attributing a new effusion to a post-pericardiotomy or non-specific postoperative process is reasonable in the first weeks after CABG. Here, however, fever, marked leukocytosis (26.3 × 10⁹/L), and lactate elevation prompted diagnostic thoracentesis, which reclassified the effusion: the fluid met two of Light's criteria for an exudate (protein ratio: 0.54; LDH ratio: 4.34), and the pH of 7.0, glucose of 29 mg/dL, neutrophilic predominance (92%), and very high nucleated-cell count together defined a complicated pleural infection requiring drainage. A postoperative attribution should therefore not preclude pleural sampling when systemic signs of infection are present. The markedly elevated D-dimer (2,749 ng/mL) fits the same theme: combined with acute hypoxemia, tachycardia, and recent surgery, it appropriately prompted CT angiography to exclude pulmonary embolism, but because D-dimer is highly sensitive and non-specific routinely elevated by recent surgery, sepsis, and acute inflammation its value here lay in justifying exclusion of embolism rather than indicating thromboembolism, and it is best read as a marker of the systemic inflammatory and postoperative state.

Management followed the two pillars of empyema care: pathogen-directed antimicrobial therapy and pleural source control. *P. multocida *is characteristically susceptible to penicillin and aminopenicillins, which are the agents of choice, with second- and third-generation cephalosporins, doxycycline, and fluoroquinolones as alternatives; first-generation cephalosporins, antistaphylococcal penicillins, clindamycin, macrolides (other than azithromycin), and aminoglycosides are unreliable, and rare β-lactamase-producing strains occur [9,13,14]. The isolate's profile (susceptible to penicillin G, ampicillin, and ceftriaxone; resistant to gentamicin) was typical. Empiric ceftriaxone plus metronidazole provided adequate coverage, and targeted de-escalation to ampicillin-sulbactam, which retains activity against possible β-lactamase-producing co-pathogens, was appropriate, with blood cultures clearing within 72 hours; the 14-day total course aligns with recommendations of 10-14 days for severe pasteurellosis [9]. The positive MRSA nasal PCR reflected colonization only, and because nasal PCR carries high negative but limited positive predictive value for MRSA pneumonia and no other evidence implicated MRSA, continued empiric anti-MRSA therapy was not warranted once *P. multocida *was confirmed. For source control, contemporary guidance favors early small-bore catheter drainage, with intrapleural tissue plasminogen activator plus DNase reserved as rescue therapy for inadequate drainage or clinical failure and surgical (video-assisted thoracoscopic surgery, VATS) referral considered in parallel for suitable candidates; in this case, chest-tube drainage plus targeted antibiotics achieved source control without intrapleural fibrinolytics or surgery [15,16].

Even with appropriate therapy, the patient deteriorated to respiratory failure on hospital Day 4, illustrating that invasive pasteurellosis with bacteremia can progress despite adequate treatment. Bacteremia and a history of neoplasia are independently associated with poor outcomes in invasive pasteurellosis (mortality ~22% in invasive disease), and bacteremia complicates a substantial fraction of *P. multocida *pneumonia [7,13]. Viewed against these risks, the patient's ultimate recovery, extubation on Day 12, a 12-day ICU stay without vasopressors, and discharge after 16 days represents a favorable outcome and reinforces the value of early source control and pathogen-directed therapy.

## Conclusions

This case describes a severe presentation of *P. multocida *empyema and bacteremia in a patient with multiple comorbidities following recent cardiac surgery. Notably, the infection occurred in the setting of household animal exposure without reported bite or scratch injury. Although the patient reported no bites or scratches, his substantial household exposure to dogs and cats suggests a zoonotic source and raises the possibility of non-bite respiratory transmission; however, the exact route of acquisition could not be established from this single case. The initial presentation closely mimicked a postoperative pleural effusion, and the subsequent progression to acute respiratory failure may have been facilitated by his underlying structural lung disease and prior thoracic radiation. This case suggests that a detailed zoonotic exposure history, including non-contact and non-traumatic exposures, may be valuable when evaluating complex or atypical pleural effusions, and that early consideration of atypical pathogens may support timely diagnosis and appropriate antimicrobial management in potentially vulnerable patients.
